# Case report: Corticosteroid-resistant acute fibrinous and organizing pneumonia with myelodysplastic syndrome

**DOI:** 10.3389/fmed.2022.1047783

**Published:** 2023-01-12

**Authors:** Dingyuan Jiang, Xueying Chen, Jun Li, Ling Zhao, Huaping Dai

**Affiliations:** ^1^National Center for Respiratory Medicine, National Clinical Research Center for Respiratory Diseases, Department of Pulmonary and Critical Care Medicine, Center of Respiratory Medicine, China-Japan Friendship Hospital, Institute of Respiratory Medicine, Chinese Academy of Medicine Sciences, Beijing, China; ^2^Laboratory Department of Pathology, China-Japan Friendship Hospital, Beijing, China

**Keywords:** acute fibrinous and organizing pneumonia, myelodysplastic syndrome, fever of unknown origin, corticosteroid-resistant, interstitial lung disease, case report, pulmonary consolidation

## Abstract

Acute fibrinous and organizing pneumonia (AFOP) is a lung disease with an unusual pathological pattern. The definitive diagnosis of AFOP relies on pathological evidence of intra-alveolar fibrin exudate, lymphoplasmacytic infiltrate, and the absence of a hyaline membrane. Furthermore, its etiology is difficult to confirm, and corticosteroids are usually effective. Herein, we report the case of a young male who presented with high fever, hemocytopenia, and consolidation in both lungs. The initial misdiagnosis was community-acquired pneumonia. Subsequently, a lung biopsy revealed abundant fibrin and fibroblast exudates in the alveolar spaces, indicating AFOP. In addition, bone marrow biopsy and karyotype analysis demonstrated that the patient simultaneously had myelodysplastic syndrome (MDS) and hemophagocytic lymphohistiocytosis. In this case, the AFOP was considered secondary to MDS; however, the disease did not respond to glucocorticoid treatment or chemotherapy. Hence, AFOP should be considered in patients with underlying hematological diseases, and early identification and diagnosis are important. Furthermore, the management of patients with severe AFOP requires further investigation.

## 1. Introduction

Acute fibrinous and organizing pneumonia (AFOP), a rare form of interstitial lung disease (ILD), is a distinct pathological manifestation of acute or subacute lung injury. AFOP pathology is characterized by intra-alveolar fibrin deposition and organizing pneumonia. The main pathological feature is described as fibrin deposition, often forming fibrin “balls,” affecting an average of 50% of alveolar spaces. Other features include a mild-to-moderate lymphoplasmacytic interstitial infiltrate, neutrophils in alveolar walls, and type II pneumocyte hyperplasia (1). The clinical presentations are non-specific, and most patients present with dyspnea, cough, and fever. Patchy or mass-like consolidation is the most common pattern in computed tomography (CT) findings. However, radiological findings could also be followed by ground glass opacity, reticular and linear opacities, and nodules (2). The prognosis of AFOP appears to have two distinct patterns: (i) an acute and unresponsive course that always leads to respiratory failure with rapid progression to death and (ii) a subacute, mild, and responsive course with recovery (3). Some AFOP cases are idiopathic, while others are induced by drugs, environmental exposure, connective tissue disease, transplantation, or tumors.

## 2. Case description

A 38-year-old male was admitted to the hospital with intermittent distressing fever lasting for 3 months. The patient was an ever-smoker (5 pack-years and had quit smoking for 1 year) with an 8-year history of thrombocytopenia. The thrombocytopenia was not diagnosed with any disease and had never been treated. The patient's blood pressure was 127/77 mmHg, heart rate was 100 beats/min, respiratory rate was 28 breaths/min, body temperature was 38.9°C, and oxygen saturation was 96% on ambient air. Soft rattles at the end of inspiration were heard in the right anterior lower chest wall. Arterial blood gas at room air revealed a pH of 7.53, partial pressure of oxygen of 74 mmHg, partial pressure of carbon dioxide of 29 mmHg, and bicarbonate of 26 mmol/L.

Three months earlier, the patient developed a fever with no apparent causes, and antifebrile treatment helped the patient return to normal body temperature, which could last for 3–5 days. One month prior, the patient was admitted to the Department of Hematology at another hospital due to a fever of unknown origin. On admission, the patient was alert and had no abnormal cardiac or abdominal findings. Chest auscultation showed no rales over the entire lung field. Laboratory tests revealed a white blood cell count of 2,800/ml, hemoglobin of 10.1 g/dl, and platelet count of 56,000/ml. The erythrocyte sedimentation rate (ESR) was 50 mm/h, C-reactive protein level 8.5 mg/dl, and ferritin 2,809 ng/ml.

**Figure 1:**
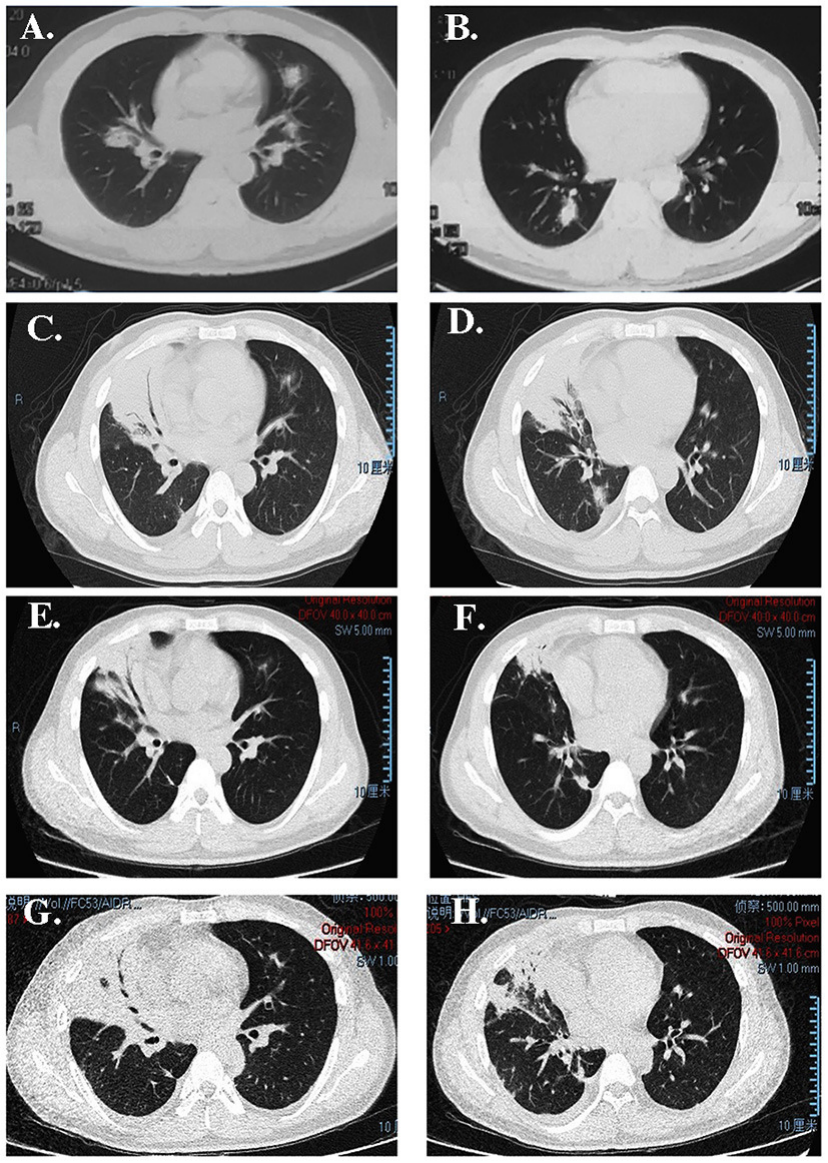
CT image showing **(A, B)** some nodules on the right upper lobe; **(C, D)** consolidation of the right upper lobe with an air bronchogram after antibiotic therapy administration in our hospital; **(E, F)** that the lesion was absorbed after treatment with dexamethasone; **(G, H)** more extensive lesions than previously observed after re-administering corticosteroids.

Additionally, alanine aminotransferase (160 IU/L), aspartate transaminase (50 IU/L), creatinine levels, and urine analysis results were normal. The antinuclear antibodies titer was 1:320, but serum immunoglobulins, complement segments, antineutrophil antibody, antineutrophil cytoplasmic antibody, and rheumatoid factor levels were all normal. Bone marrow biopsy showed that the bone marrow hyperplasia was generally normal, and the hematopoietic cells of granulocytes showed hyperplasia, no primitive cells, and abnormal lymphocytosis. On chest CT, small nodules and small patchy consolidations were observed in the right lung, while patchy consolidations appeared in the left upper lung lobe and basement of the left lower lung (Figures 1A, B). The patient had negative blood and sputum cultures and received broad-spectrum antibiotics (cefoperazone/tazobactam was initially administered, then switched to moxifloxacin and fluconazole intravenously) for almost 1 month. The fever did not improve, and the patient started to experience slight chest pain on the right side and sometimes had sputum with little blood.

On admission to our hospital, laboratory data revealed a white blood cell count of 1,630/ml (neutrophil 970/ml and lymphocyte 630/ml), hemoglobin of 5.5 g/dl, platelet count of 75,000/ml, ESR of 140 mm/h, and serum ferritin levels of 2,037 ng/ml. The cluster of differentiation 4 and 3 (CD4^+^CD3^+^) lymphocyte count was 230 cells/μl, and Coombs test results were negative. Furthermore, the serum Krebs von den Lungen-6 level was 865 U/ml, and all autoimmune test results were negative.

**Figure 2:**
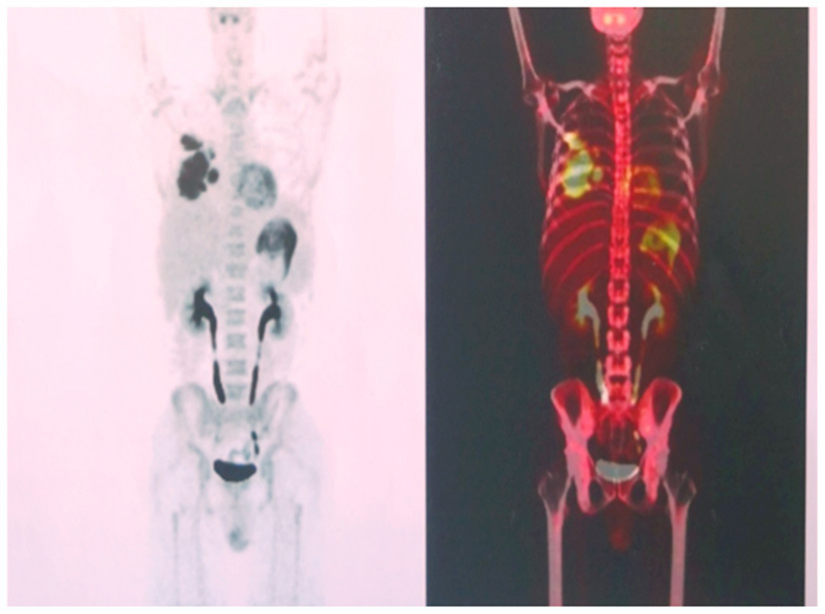
High levels of 18F-fluorodeoxyglucose uptake on the right lung observed on positron emission tomography.

The microbial infection was thoroughly examined *via* culture and smear of the sputum, blood, and bronchoalveolar lavage fluid (BALF). Only Epstein-Barr virus nucleic acid was detected in the sputum and BALF samples. The scope of pulmonary consolidation on chest CT was significantly larger than that before (Figures 1C, D). Consolidations and nodules in the right lung showed higher levels of 18F-fluorodeoxyglucose (18F-FDG) uptake on positron emission tomography (PET) (Figure 2) with a maximum standardized uptake value (SUV_max_) of 10.1. The patch in the left lower lobe had an SUV_max_ of 2.3, and 18F-FDG uptake also increased in several mediastinal lymph nodes with an SUV_max_ of 3.7, suggesting that the nature of the lung impairment might have been malignant.

The patient began to experience a distressing fever of up to 40°C daily, and antifebrile treatment could only help sustain a normal body temperature for 4–6 h. Since the patient had a high fever and high levels of ferritin and cytopenia, a test was performed for the soluble CD25 (sCD25) level, which was 9,409 pg/ml, and the activity of natural killer cells was 12%. The patient met five of the eight factors comprising the hemophagocytic lymphohistiocytosis (HLH) 2004 Diagnostic Criteria and was diagnosed with hemophagocytic syndrome (HPS) (4). However, the bone marrow smear did not show any signs of hemophagy. The complete therapeutic regimen for HPS with etoposide and cyclosporin A was not administered because HPS could not explain the whole presentation, and the patient was immunosuppressed. Dexamethasone was administered intravenously at a dose of 10 mg daily, immunoglobulin was administered intravenously, and further diagnostic investigations continued. During dexamethasone treatment, the patient's body temperature was normal for several days, and the right lung consolidation appeared small (Figures 1E, F). Unfortunately, the patient developed a high fever as soon as the dexamethasone dose was decreased to 5 mg, according to the therapeutic regimen for HPS.

**Figure 3:**
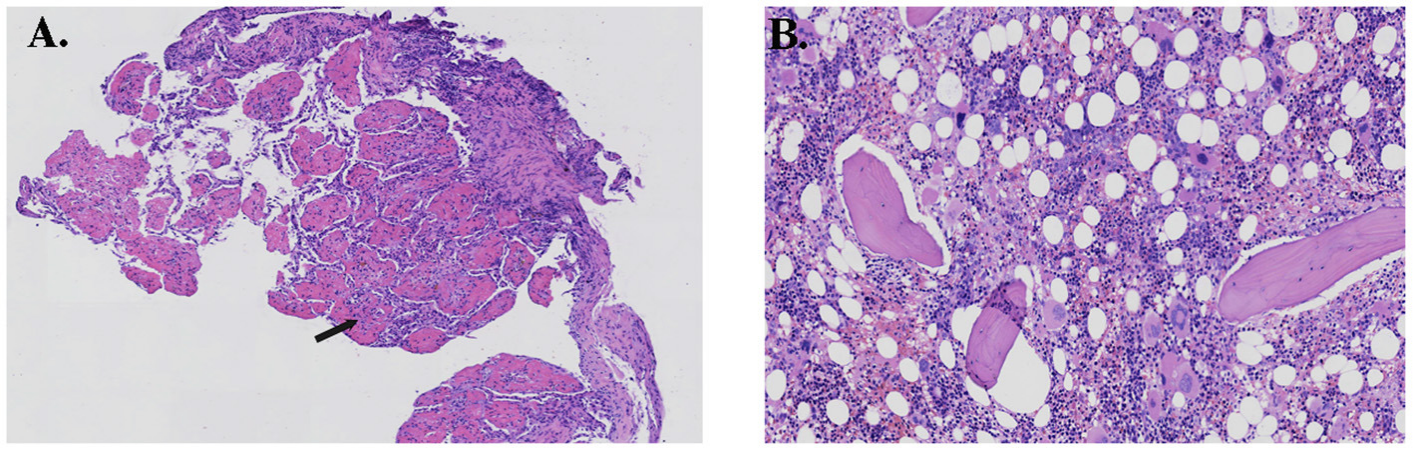
**(A)** Histopathology of the lung showing multiple organizing “fibrin balls” (arrow) within alveolar spaces, and **(B)** histopathology of the bone marrow showing active hyperplasia.

Finally, after a percutaneous fine needle biopsy of the right lung mass confirmed AFOP, alveolar spaces were filled with abundant inflammatory fibrin exudate with an immature organization (Figure 3A). On execution of bone marrow biopsies, active bone marrow hyperplasia was observed, and the number of immature cells in the medullary cavity was significantly increased (Figure 3B). The patient had a mutation in the ASXL1 gene, and the karyotype analysis revealed 48, XY, +8, +16, der (19) t (1, 19) (q12; p13) [19]/49, idem, +20 [1]. Myelodysplastic syndrome (MDS) was diagnosed based on all the results given above and defined as MDS with multilineage dysplasia (MDS-MLD) with the Revised International Prognostic Scoring System score of 6, which was “high.”

**Figure 4:**
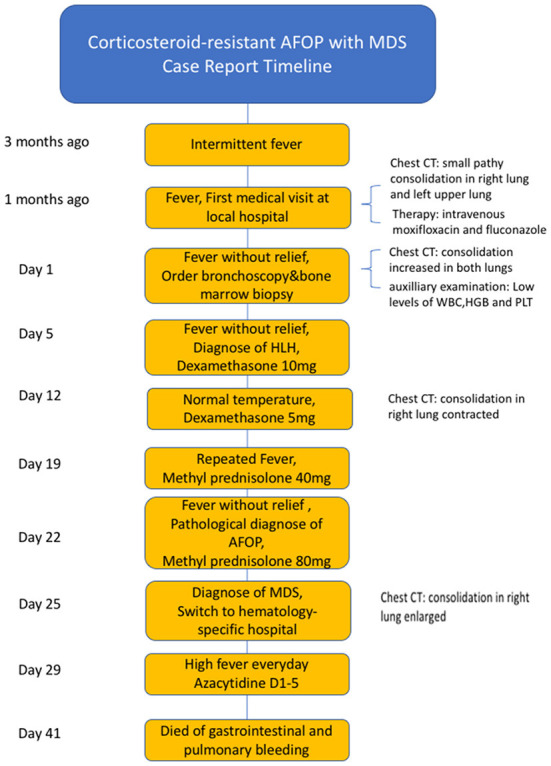
The timeline of the case.

Considering the AFOP, intravenous methylprednisolone (80 mg) and immunoglobulin were re-administered daily. However, the fever did not subside, and the consolidation on the CT image progressed (Figures 1G, H). The patient's oxygen saturation was 96% on ambient air, and there was no incidence of respiratory failure. The patient was admitted to a hematology-specific hospital for further MDS treatment, and both broad-spectrum antibiotics and methylprednisolone were administered. However, there was no further improvement in lung impairment. Moreover, the patient still experienced a high fever daily. Finally, a decision was made to administer azacitidine as chemotherapy for MDS. Unfortunately, the patient did not show any improvement in the distress fever and eventually died of gastrointestinal and pulmonary bleeding due to bone marrow suppression resulting from chemotherapy. The progress and decision-making involved in this case are shown in the timeline (Figure 4).

## 3. Discussion

This case demonstrates that lung infiltration may be the onset manifestation of MDS in patients with a first-time MDS diagnosis. In addition, AFOP should be considered as a differential diagnosis when MDS complications with pulmonary consolidation are refractory to antibiotics. Although a lung biopsy eventually demonstrates pulmonary impairment in AFOP and corticosteroids have always been reported to be effective, in this case, corticosteroid administration persistently did not improve either the fever symptoms or pulmonary consolidation. Furthermore, chemotherapy for MDS posed a high risk for severe infection in this case, considering the pulmonary infiltrations and continuous fever. Corticosteroids and antibiotics were administered first to help improve the overall situation; however, these therapies failed. Azacytidine for MDS treatment did not affect the patient's quality of life. Simultaneous diagnoses of MDS and AFOP are rare, and none of the previous cases reported high-risk MDS with corticosteroid-resistant AFOP. Both MDS and AFOP were intractable in these patients, and managing these two diseases is still a big challenge.

There are a few case reports of patients with MDS who had pulmonary infiltrates and were diagnosed with AFOP. In these cases, MDS is always diagnosed first; hence, AFOP is considered to be induced by drugs administered for MDS treatment. Moreover, some other case reports considered that AFOP was caused by MDS-related autoimmune responses (5–10). The patients in these cases were aged 49–72 years, and the duration between AFOP and MDS diagnoses varied from 2 months to 2 years. AFOP has been described as fever, cough, and respiratory failure in a few cases. Additionally, its description on CT scan images varies in different cases; it could involve both lungs or any of them, presenting as consolidation, ground-glass opacities, or multiple micronodules.

Corticosteroids were reported to be effective in all the reported cases of AFOP above. Immunosuppressive agents have also been suggested and frequently result in good responses to therapy. AFOP was treated with corticosteroids in all these reported cases, and the onset dose varied from 1 g to 40 mg intravenously. All patients responded well, with symptom improvements, including clinical presentations such as fever and dyspnea and alleviation of radiological findings. Although some patients had recurrent symptoms during corticosteroid dose reduction or recurrence with MDS deterioration, patients with AFOP can still benefit from corticosteroid re-administration. This report presents a case of MDS manifesting as AFOP. This case is special because AFOP had a poor response to corticosteroids, the symptoms did not improve, and the disease persistently worsened despite administering corticosteroids and chemotherapy for MDS.

Acute fibrinous and organizing pneumonia is often misdiagnosed as pneumonia, tuberculosis, or malignancy due to a lack of typical clinical manifestations. The patient, in this case, presented with symptoms of community-acquired pneumonia, fever, and an elevated ESR. The symptoms did not improve after regular anti-infective treatment, and the pulmonary lesions were more severe than before. Hence, tuberculosis and malignancy were considered; this prompted us to consider bronchoscopy to obtain a tissue diagnosis and seek evidence of the infectious pathogen from the BALF and lung tissue. Repeated high fever with blood cytopenia prompted the consideration of PET-CT and bone marrow biopsy to obtain the disease involvement of other body organs and evidence for the possibility of hematological disease diagnosis.

Acute fibrinous and organizing pneumonia is a rare interstitial pulmonary disease. It can be idiopathic or associated with underlying diseases or conditions, such as connective tissue or immune system disorders, infections, drug administration, and occupational exposure (3, 11). AFOP is considered a histological pattern in the clinical spectrum of diffuse alveolar damage and organizing pneumonia. Patchy or mass-like consolidation is the most common pattern of AFOP and appears to have a better prognosis (2). In addition to consolidation and nodules, CT findings of AFOP could also present as ground-glass opacity and reticular or linear opacity. Moreover, idiopathic etiology, fever, and immunosuppressive therapy have been significantly associated with survival in patients with AFOP. In some studies, the overall mortality rate was ~6–54% (3, 12–14), which may be due to the heterogeneous phenotype of AFOP. Most patients who recovered from AFOP maintained a normal life without supplemental oxygen therapy or respiratory symptoms (13). Although the patient, in this case, was in a sub-acute course and had a fever and mass-like consolidation, the poor prognosis may be related to the underlying MDS.

ILDs are not rare in patients with MDS; a cohort study including 827 patients with MDS reported that 2% of the patients had an ILD. Furthermore, ILD was diagnosed before MDS in 72% of the patients and was present in a higher percentage than anticipated in the population of patients with MDS (15). ILD development is thought to be linked to autoimmune dysregulation as a result of MDS or as a result of immune-modulating treatments. Immune suppression and anti-inflammatory therapy are usually attempted for both low-risk MDS and many ILDs. The exact mechanism of ILD in MDS is still unknown, and the pathogenesis of these two diseases requires further investigation.

Azacitidine therapy is the main treatment for MDS and has been linked to ILD development, with direct tissue injury and the creation of a profibrotic milieu (15). Lin et al. (6) reported a case in which MDS turned into acute myeloid leukemia, with simultaneous recurrence of AFOP. Another course of high-dose prednisone with immunosuppressive therapy improved the symptoms. Hence, it seems that AFOP might develop in patients with MDS. However, there are no clues as to whether the treatment of MDS will effectively control AFOP. In the present case, AFOP was diagnosed before initiating azacytidine therapy. However, azacytidine did not help control either MDS progression or AFOP, resulting in a deteriorated situation.

Corticosteroids are still the critical treatment for AFOP, and most patients with AFOP show a good response to initial corticosteroid treatment and re-administration for recurrence (16). However, there are no established recommendations regarding dosing and treatment duration, ranging from low doses to boluses of 250–1,000 mg for 3 days (12, 17). The regimen is usually prolonged for months or years to avoid recurrence, both in idiopathic cases and cases secondary to autoimmune diseases. In addition, immunosuppressants such as mycophenolate mofetil, azathioprine, and cyclophosphamide can be used in both acute and maintenance phases (18).

Some studies have suggested that the response to treatment depends on the severity of the underlying disease (19, 20), especially hematological and autoimmune diseases. However, no cases of corticosteroid-resistant AFOP combined with MDS have been reported.

The present case is unique because AFOP appeared simultaneously with a hematological abnormality that was finally diagnosed as MDS. Bone marrow biopsy was performed early to determine the reason for hypocytosis; however, there were no apparent abnormalities in the early stages of the disease that could help confirm the underlying MDS. Hence, gene and karyotype analyses are key clues for MDS diagnoses. AFOP, in this case, had a rapid progression, no improvement in response to glucocorticoids, and a poor prognosis. Neither immunoglobulin nor corticosteroid re-administration at an increased dose helped with lung impairment. The delayed diagnosis of MDS might have contributed to the rapid progression of AFOP and poor response to therapy; MDS combined with AFOP can progress rapidly.

In conclusion, we report a case of MDS with non-infectious pulmonary complications, namely, AFOP. ILDs, such as AFOP, should be prioritized in patients with MDS or other hematological and autoimmune diseases, especially in patients that do not respond to antibiotic treatments. The two diseases are intertwined, making their diagnosis and treatment difficult. AFOP in these patients was resistant to corticosteroid treatment, and MDS treatment had no beneficial outcome either. The patient eventually died from multi-organ bleeding due to myelosuppression. Hence, immunosuppressive agents may be worth investigating. There are still unknown questions about AFOP; therefore, more researchers need to pay attention to this disease.

## Data availability statement

The original contributions presented in the study are included in the article/supplementary material, further inquiries can be directed to the corresponding author.

## Ethics statement

Written informed consent was obtained from the individual(s) for the publication of any potentially identifiable images or data included in this article.

## Author contributions

The first draft of the manuscript was produced by DJ, XC, and HD. DJ, XC, and JL were the major contributors to the analysis and interpretation of patient data. DJ and HD were the major contributors to the writing of the manuscript. In addition, LZ performed a histological examination of the patient's lungs. All authors have reviewed, edited, and approved the final version of the manuscript.
